# Diarrhea as the Initial Presentation in a Patient With HIV Diagnosed With Hodgkin’s Lymphoma

**DOI:** 10.7759/cureus.61361

**Published:** 2024-05-30

**Authors:** Nicol Tugarinov, Esther Xu, Grace H Kim

**Affiliations:** 1 Department of Medicine, University of Maryland School of Medicine, Baltimore, USA; 2 Department of Medicine, Mercy Medical Center, Baltimore, USA

**Keywords:** hiv lymphoma, persistent diarrhea, human immunedeficiecy virus (hiv) infection, gastrointestinal symptoms, classic hodgkin lymphoma

## Abstract

Hodgkin’s lymphoma (HL) is a form of cancer that involves abnormal lymphocyte proliferation which affects the lymphatic system. Patients with HIV are at increased risk of developing HL, despite the introduction of combination antiretroviral therapy. The most common presentation of HL is painless lymphadenopathy with classic constitutional symptoms in advanced disease. Here we discuss a 39-year-old female with a history of HIV on emtricitabine/tenofovir and dolutegravir who presented with four days of worsening diarrhea along with fevers and chills. She had a similar presentation at a nearby hospital four months prior. After initial concern for gastrointestinal infection, an extensive infectious workup was conducted and was negative. After complaints of sore throat and increased confusion during the hospital stay, a CT Chest and Neck revealed diffuse lymphadenopathy. Severely elevated ferritin levels raised concern for secondary hemophagocytic lymphohistiocytosis and prompted expedited ultrasound-guided cervical lymph node (LN) core biopsy and bone marrow biopsy. Ultrasound-guided core biopsy of the LN showed classical Hodgkin’s lymphoma of mixed cellularity. The patient was started on doxorubicin, vinblastine, and dacarbazine + nivolumab. This is a case of a patient with HIV who presented with chronic diarrhea of unidentifiable origin and was ultimately diagnosed with classical Hodgkin’s lymphoma during her hospitalization and highlights the importance of maintaining lymphoproliferative diseases on the differential in patients with HIV and gastrointestinal symptoms.

## Introduction

Hodgkin’s lymphoma (HL) is a rare neoplasm characterized by monoclonal proliferation of lymphocytes. The two subtypes of HL are classical HL and nodular-lymphocyte predominant HL. Classical HL accounts for the majority of cases and is composed of four distinct subgroups: nodular sclerosis (NSHL), lymphocyte-rich (LRHL), mixed cellularity (MCHL), and lymphocyte-depleted (LDHL) [1]. NSHL and MCHL account for the largest percentage of classical HL cases [2]. Patients living with human immunodeficiency virus (HIV) are five to 26 times more likely to develop Hodgkin’s lymphoma compared to HIV-negative patients [3]. Additionally, the risk of HL has increased since the introduction of combined antiretroviral therapy (cART) with possible association with CD4 T-cell count [4]. Common presentations of HL include painless lymphadenopathy, most commonly involving cervical, axillary, mediastinal, and supraclavicular nodes [5]. Constitutional symptoms (i.e. fever, night sweats, unexplained weight loss > 10% of body weight) are more commonly seen in patients with advanced disease and can have prognostic value [6]. Atypical presentations of HL have been reported and are more common in patients with HIV or other immunosuppressive conditions [7]. Treatment of HL is generally centered around chemotherapy and radiation. Due to improvements in the understanding of disease mechanisms of HL, the five-year survival rate of HL is 89.8% for those diagnosed between the ages of 20-64, making it a highly curable malignancy [8]. Additionally, several studies have shown that although HIV-positive patients tend to have more aggressive characteristics of classical HL, there is no difference in response rate or survival in comparison to HIV-negative patients [9,10]. Thus, it is important to be able to identify patients with uncommon presentations of HL for timely initiation of treatment due to the high potential for recovery.

Here we discuss a case of a patient with HIV who had persistent diarrhea as their initial presenting symptom and was subsequently diagnosed with Hodgkin's lymphoma. We aim to raise awareness of possible atypical presentations of classical HL, especially in immunosuppressed populations.

## Case presentation

**Figure 1:**
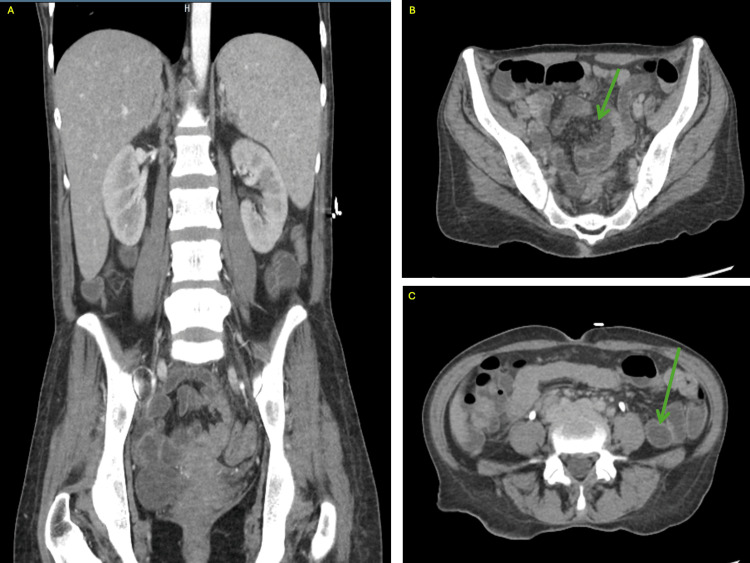
Computed tomography imaging of the abdomen and pelvis showing hepatosplenomegaly and non-specific enteritis A:Coronal view demonstrating substantial hepatosplenomegaly. B-C: Axial views demonstrating infectious versus inflammatory enteritis.

A 39-year-old woman with HIV on emtricitabine/tenofovir and dolutegravir presented with four days of worsening diarrhea with fevers and chills. She had a similar presentation at a nearby hospital four months prior for a month of diarrheal symptoms. Previous infectious stool studies were negative and CT imaging of the abdomen at the time showed nonspecific retroperitoneal and upper mesenteric lymphadenopathy with no significant bowel wall thickening or pericolonic stranding and liquid stool throughout the colon and rectum. The discharge diagnosis was diarrhea secondary to presumed infectious gastroenteritis. Since her prior hospitalization, she reported lasting diarrhea with inability to tolerate solid foods, continued intermittent fevers with temperatures ranging from 101 - 104 degrees Fahrenheit, and weight loss. She described her diarrhea as yellow/green-colored and watery, and denied any associated nausea or vomiting. She endorsed having over three bowel movements per day. Due to concern for an infectious etiology and the chronicity of her symptoms in the setting of her known HIV diagnosis, she underwent an extensive workup on presentation, which included computerized tomography (CT) of the abdomen and pelvis with intravenous contrast, an infectious gastrointestinal (GI) pathogens panel, blood cultures, cytomegalovirus (CMV) DNA quantification, lipase, complete blood count, complete metabolic panel, coagulation studies, and HIV RNA quantification. The infectious GI pathogens panel, which includes bacterial, viral and protozoal pathogens, and CMV DNA quantification returned negative and the lipase level was within normal limits (39 IU/L). CT of the abdomen and pelvis on admission was notable for inflammatory versus infectious enteritis and hepatosplenomegaly (Figure 1). Basic laboratory values are displayed in Table 1. 

**Table 1:** Basic laboratory values obtained on day one of hospitalization ND: not detected, BUN: blood urea nitrogen, ALT: alanine aminotransferase, AST: aspartate aminotransferase, Abs: antibodies

Lab Parameters	Patient Value	Reference Range
White Blood Cell Count (10^3/uL)	6.1	4.5-10.5
Red Blood Cell Count (10^6/uL)	2.77	3.7-5.3
Hemoglobin (g/dL)	7.8	12.0-15.5
Hematocrit (%)	24	36.0-46.0
Platelets (10^3/uL)	114	150-450
Segmented Neutrophils (%)	39.7	35.0-70.0
Bands (%)	24.1	0.0-5.0
Lymphocytes (%)	16.4	15.0-55.0
Reactive Lymphocytes (%)	3.4	0.0-5.0
Monocytes (%)	13.8	0.0-10.0
Metamyelocytes (%)	1.7	0.0-1.0
Absolute Reactive Lymphocytes (10^3/uL)	0.21	0.0-0.0
Absolute Metamyelocytes (10^3/uL)	0.1	0.0-0.05
Sodium (mEq/L)	127	136-145
Potassium (mEq/L)	4.3	3.5-5.3
Chloride (mEq/L)	96	98-107
CO2 (mEq/L)	16	22-32
Anion Gap	15	5-17
BUN (mg/dL)	21	8-23
Creatinine (mg/dL)	1.2	0.5-1.0
Glucose (mg/dL)	116	65-100
Calcium (mg/dL)	8.4	8.5-10.5
Magnesium (mg/dL)	2	1.6-2.4
Alkaline Phosphatase (IU/L)	114	30-126
Albumin (g/dL)	3.7	3.2-5.0
AST (IU/L)	35	8-35
ALT (IU/L)	12	10-40
Total Bilirubin (mg/dL)	0.6	0.2-1.4
Prothrombin (seconds)	16.7	11-15
International Normalized Ratio (INR)	1.3	0.0-4.0
Partial Thromboplastin Time (seconds)	57.7	24-36
CD3 Abs (/uL)	265	622-2402
CD3 % Total T cell	37.8	57.5-86.2
CD4 T cell Abs (/uL)	175	359-1519
CD4 % Helper T cell	25	30.8-58.5
CD4/CD8 Ratio	1.88	0.92-3.72
CD8 T cell Abs	93	109-897
CD8 % Suppressor T cell	13.3	12.0-35.5
Absolute Lymphocytes (10^3/uL)	0.7	0.7-3.1
Lactic Acid (mmol/L)	3.9	0.7-2.1

**Figure 2:**
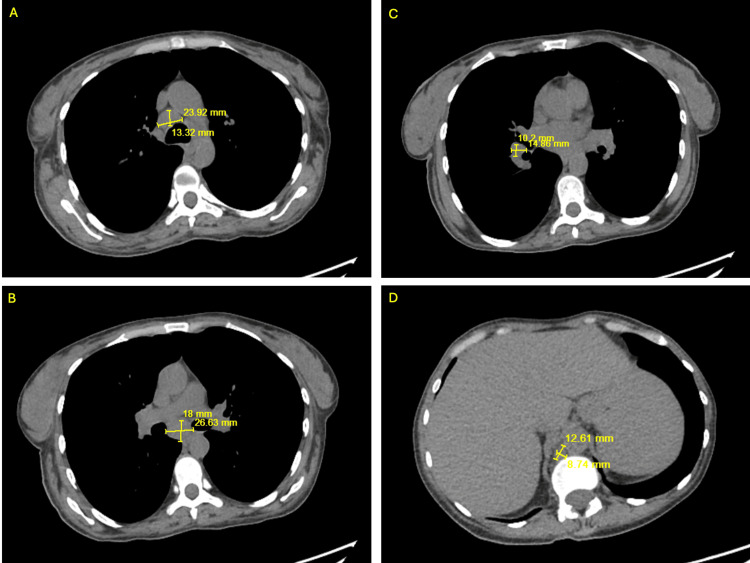
Computed tomography imaging of the chest demonstrating mediastinal and upper abdominal lymphadenopathy A-C: Axial view of multiple mediastinal lymph nodes. D: Axial view of upper abdominal adenopathy.

**Figure 3:**
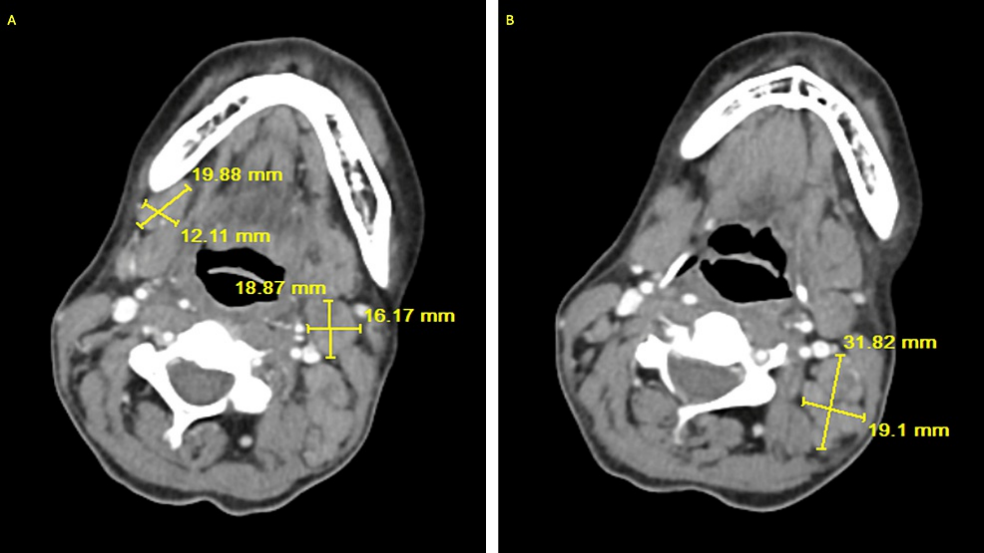
Computed tomography imaging of the soft tissue of the neck with marked cervical lymphadenopathy

Infectious disease was consulted on hospital day two who recommended ova and parasite stool studies. The patient reported strict compliance to her cART. Her CD4 T cell count five months prior to admission was 845 with an undetectable viral load. A repeat CD4 count during this admission was 175 and her HIV RNA load was undetectable. An esophagogastroduodenoscopy (EGD) and colonoscopy was planned on hospital day six, however it was never started due to concerns for sepsis due to recurrent fevers while in the endoscopy suite (TMax of 101.6 Fahrenheit) and an elevated lactate level. A sepsis workup was initiated and was found to be unremarkable. On hospital day eight, the patient complained of a sore throat with increased confusion. Non-contrast CT of the head showed no signs of an acute intracranial pathology. CT of the chest showed numerous small and mildly enlarged supraclavicular, pre-pectoral, axillary, mediastinal, hilar, and upper abdominal adenopathy (Figure 2). CT of the soft tissue of the neck with contrast showed cervical lymphadenopathy, most pronounced on the left side (Figure 3).

Hematology and Oncology was consulted at this time due to concern for malignancy, who recommended a lymph node (LN) biopsy to evaluate for lymphoproliferative disorder. Notable hematologic labs throughout her hospital course are displayed in Table 2. 

**Table 2:** Hematologic laboratory values obtained throughout hospitalization IL-2: interleukin-2; NM: not measured

Lab Parameter	Day 2	Day 12	Day 14	Day 15	Reference Range
Ferritin (ng/mL)	NM	NM	9,968	10,334	12-150
Iron Level (mcg/dL)	39	12	NM	NM	60-170
Iron Saturation (%)	18.4	8.2	NM	NM	20-50
Total Iron Binding Capacity (ug/dL)	212	146	NM	NM	240-450
Lactate Dehydrogenase (U/L)	716	532	NM	NM	140-280
Haptoglobin (mg/dL)	NM	24	NM	NM	41-165
Absolute Reticulocytes (10^6/ul)	0.0319	0.0106	NM	0.0207	0.023-0.1
Reticulocyte Count (%)	1.17	0.49	NM	0.72	0.5-2.17
Reticulocyte Hgb Equivalent (pg)	22.0	21.8	NM	22.0	28-36
Immature Reticulocyte Fraction (%)	10.6	6.6	NM	21.3	2.3-13.4
Serum Soluble IL-2 Receptor (pg/mL)	NM	NM	NM	39,345.6	175.3-858.2

Her diarrhea resolved on hospital day 14 with symptomatic treatment with loperamide 2 mg as needed which was initiated only after the infectious GI panel returned negative. Ferritin levels were ordered to characterize continued normocytic anemia, which came back elevated (>9000 ng/mL). This raised concern for secondary hemophagocytic lymphohistiocytosis and prompted expedited ultrasound-guided cervical LN biopsy and bone marrow biopsy, along with fungal and acid-fast bacilli (AFB) cultures. Biopsy of the LN showed classical HL of mixed cellularity (CD10 negative). Bone marrow biopsy showed extensive involvement of the patient’s known classical Hodgkin’s lymphoma. Fungal and AFB cultures of the bone marrow were negative.

**Figure 4:**
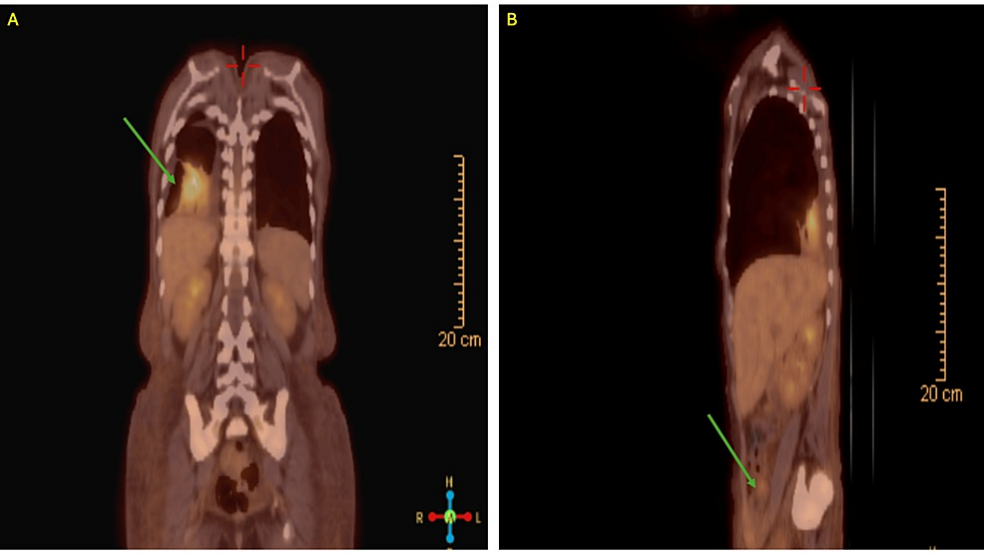
Positron emission tomography imaging for initial staging of Hodgkin's lymphoma A: Coronal view of the right lower lobe consolidation with intense uptake, B: Axial view of hepatomegaly and inguinal/retroperitoneal lymphadenopathy with minimal uptake.

After diagnosis of her classical HL, she was started on doxorubicin, vinblastine, and dacarbazine + nivolumab. An F18 fluoro-D-glucose positron emission tomography (FDG-PET) CT scan was performed for initial staging which showed multiple sub-centimeter neck, axillary, sub-pectoral and mediastinal lymph nodes with minimal metabolic activity, intense metabolic activity in a consolidation in the right lower lobe concerning for pneumonia, and sub-centimeter retroperitoneal and inguinal lymph nodes with minimal metabolic activity, however evaluation of the abdomen and pelvis was limited due to lack of intravenous and oral contrast administration (Figure 4).

## Discussion

We discuss a 39-year-old woman with HIV who presented with chronic diarrhea and was ultimately diagnosed with classical HL from cervical LN and bone marrow biopsy. The cause of her diarrhea could not be ascertained, however, it is possible that her diagnosis of Hodgkin's lymphoma contributed to her symptoms given prior reports of Hodgkin's lymphoma presenting with gastrointestinal symptoms [11,12]. While the most common presentation for HL is painless lymphadenopathy, her presentation and hospital course were complex, delaying the diagnosis of lymphoma. Patients with HIV can also experience diarrhea due to noninfectious causes such as ART-associated diarrhea or HIV-associated enteropathy. With her low CD4 T cell counts at presentation, concern for opportunistic infection was high, despite her undetectable viral load. Given this concern, numerous extensive infectious stool studies were performed, all of which returned negative. Given the chronicity of her diarrhea and negative results of two infectious GI panels within a six-month period, it is less likely that her diarrhea was due to an infectious cause. Her lymphadenopathy was incidentally discovered on CT imaging after she complained of a worsening sore throat. Patients with HIV are at increased risk of developing Hodgkin’s lymphoma, with the mixed cellularity subtype having increasing prevalence with the introduction of cART [4]. Suspicion should remain high in this patient population despite seemingly atypical clinical presentations.

This patient presented with a declining CD4 count and an unchanged viral load. Although her CD4 count being < 200 raises concern for the development of opportunistic infection, her viral load remained undetectable. Patients with HIV on cART may still contract opportunistic infections; however, their laboratory values typically show a decrease in CD4 count with a subsequent increase in HIV viral load [13]. Therefore, this patient’s declining CD4 count was more likely attributed to a hematological pathology. Although we were not able to rule out other pathologies such as celiac disease and microscopic colitis, which can also present with chronic diarrhea, their diagnoses are less likely given the lack of associated symptoms such as flatulence and bloating, and iron deficiency anemia in the case of celiac disease. Additionally, these patients are less likely to present with fevers and weight loss. Finally, given the patient's immunosuppressed status, it is highly unlikely she would have a concomitant autoimmune condition such as celiac disease. Although the patient's staging FGD-PET/CT showed subcentimeter retroperitoneal and inguinal lymphadenopathy with minimal uptake, this study was limited due to the lack of intravenous or oral contrast and does not help with identification of a primary lesion in the gastrointestinal tract that could have caused the patient's symptoms. A colonoscopy with biopsies of the bowel would have been incredibly useful for diagnosis and rule out of other conditions, which is a major limitation of this workup.

In addition to abnormal cell counts, other hematologic abnormalities seen in this patient included a significantly elevated ferritin level. Combined with diffuse lymphadenopathy, urgent hematologic exploration was initiated to evaluate for possible malignancy or hemophagocytic lymphohistiocytosis (HLH). HLH is a life-threatening syndrome associated with hemophagocytosis of red blood cells, neutrophils, and platelets by activated macrophages [14]. HLH is difficult to diagnose, and extreme hyperferritinemia is a hallmark sign. Our patient had an extremely high ferritin level, along with other indicators of disease (i.e fever, hepatosplenomegaly on imaging, severe anemia, elevated serum soluble IL-2 receptor levels). It is likely that she exhibited a component HLH; however, her hyperferritinemia could also be attributed to other factors. Her final diagnosis of Hodgkin’s lymphoma is associated with high ferritin levels, as ferritin is one of the tumor-associated antigens in this disease [15].

## Conclusions

This is a novel case of a patient with HIV who presented with chronic diarrhea as their initial symptom and was ultimately diagnosed with classical Hodgkin's lymphoma. Although the exact cause of the diarrhea could not be ascertained, it is likely attributable to her diagnosis of Hodgkin's lymphoma given the negative infectious work-up and prior reports of patients with Hodgkin's lymphoma presenting with gastrointestinal symptoms. Although our patient did not undergo an EGD/colonoscopy while she was hospitalized, it could have provided worthwhile information as to if there was any identifiable mass or lesion that could explain the atypical presentation of this case. This report provides a unique case of a patient with HIV presenting with gastrointestinal symptoms of unidentifiable origin who was ultimately diagnosed with Hodgkin's lymphoma and emphasizes the importance of maintaining lymphoproliferative diseases on the differential in patients with HIV with gastrointestinal symptoms.
